# Comprehensive Geriatric Care in Older Adults: Walking Ability after an Acute Fracture

**DOI:** 10.3390/medsci11020040

**Published:** 2023-05-29

**Authors:** Ulrich Niemöller, Andreas Arnold, Thomas Stein, Martin Juenemann, Damir Erkapic, Josef Rosenbauer, Karel Kostev, Marco Meyer, Christian Tanislav

**Affiliations:** 1 Department of Geriatrics and Neurology, Diakonie Hospital Jung Stilling Siegen, 57074 Siegen, Germany; 2 Department of Neurology, Justus Liebig University, 35392 Giessen, Germany; 3 Department of Cardiology, Diakonie Hospital Jung Stilling Siegen, 57074 Siegen, Germany; 4 Department of Epidemiology, Philipps University Marburg, 35043 Marburg, Germany

**Keywords:** comprehensive geriatric care, elderly patients, fractures, outcome

## Abstract

Background/Objectives: Comprehensive Geriatric Care (CGC) is a specific multimodal treatment for older patients. In the current study, we aimed to investigate walking performance after CGC in medically ill patients versus those with fractures. Methods: The timed up and go test (TuG), a 5-grade scale assessment (1 = no walking impairment to 5 = no walking ability at all) for evaluating individual walking ability was performed in all patients who underwent CGC prior to and after treatment. Factors associated with improvement in walking ability were analyzed in the subgroup of patients with fractures. Results: Out of 1263 hospitalized patients, 1099 underwent CGC (median age: 83.1 years (IQR 79.0–87.8 years); 64.1% were female). Patients with fractures (*n* = 300) were older than those without (*n* = 799), (median 85.6 versus 82.4 years, *p* = 0.001). Improvement in TuG after CGC was found in 54.2% of the fracture patients compared to just 45.9% of those without fractures. In fracture group patients, TuG improved from median 5 on admission to median 3 on discharge (*p* = 0.001). In fracture patients, improvement in walking ability was associated with higher Barthel index values on admission (median 45 (IQR: 35–55) versus 35 (IQR: 20–50): *p* = 0.001) and Tinetti assessment scores (median 9 (IQR: 4–14.25) versus 5 (IQR: 0–13); *p* = 0.001) and was negatively associated with the diagnosis of dementia (21.4% versus 31.5%; *p* = 0.058). Conclusion: CGC improved walking ability in more than half of all patients examined. Older patients in particular might benefit from undergoing the procedure after an acute fracture. A better initial functional status favors a positive result following the treatment.

## 1. Background

Geriatric departments have been established in many countries for the treatment of elderly patients. In accordance with standardized protocols, treatment regimens follow specific recommendations focusing on medical requirements but also taking into account functional outcomes [1,2,3]. Comprehensive geriatric care (CGC) is a multimodal care approach facilitating structured treatment for hospitalized elderly patients with various defined disorders [4,5,6,7,8,9]. Multidisciplinary discussion as a team including different medical professionals such as occupational therapists, physiotherapists and speech therapists, dietitians, psychologists, or social workers aimed at determining the best treatment strategy for an individual patient forms the core of CGC [10,11,12,13,14,15,16,17]. The literature reviewed investigated heterogeneous populations undergoing CGC, addressing medical, surgical, or patients with neurological disorders [6,18,19,20,21,22,23,24]. Patients with fractures after falls are of particular relevance in the more recent literature [25]. Some evidence indicates that CGC is effective in improving the functional outcome in elderly patients after falls and/or fractures [26,27]. However, there is a lack of investigations aimed at demonstrating the benefits of CGC in a routine care environment. Conditions in the routine clinical settings in which caregivers work are different from the strict pre-specified workflows and rigorous selection of patients that prevail in clinical trials [28,29]. The positive effects of CGC, therefore, require verification by means of large-scale investigations [30,31,32].

It is for this reason that we investigated walking performance in older patients after CGC in a large department for geriatrics in the present study.

## 2. Methods

### 2.1. Patients and Measures

**Figure 1:**
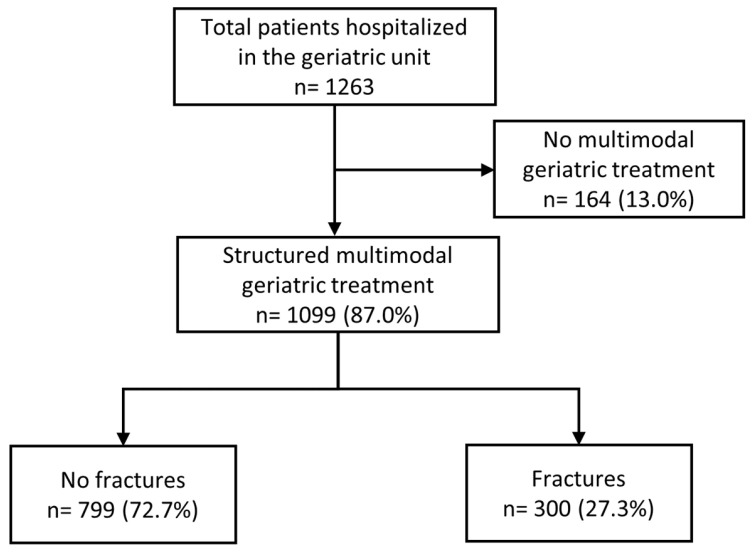
Patient selection.

All patients hospitalized between May 2018 and May 2019 were selected. The main inclusion criterion for the present analysis was the completion of CGC within this period; the subset of patients with complete documentation was included in the analyses (Figure 1).

Patients were referred to our department for CGC from the emergency department or from other external or in-house departments and general practitioners. After a detailed assessment, patients were referred for CGC where appropriate. A standardized geriatric assessment regarding patients’ mobility, ability to cope with daily tasks, cognitive function, and emotional and social condition was performed on hospital admission and also on discharge. CGC was defined as a multi-component intervention addressing multiple health domains to develop a person-centered therapeutic plan satisfying acute medical requirements and rehabilitation needs. The selected treatment regime was adapted to reflect patients’ deficits and was continuously re-evaluated. CGC included treatment by an interdisciplinary team consisting of geriatric nursing, physiotherapists, occupational therapists, speech therapists, and psychologists under the supervision of an experienced geriatrician. A minimum of 20 regular treatment units of physiotherapy and/or occupational therapy were scheduled for each patient. According to the CGC protocol, a minimum period of 2 weeks was allocated to complete the required therapy units. Medical visits were carried out daily by a geriatrician; medications were adapted and diagnostic procedures were undertaken if necessary. Team conferences took place weekly on the basis of a standardized protocol to discuss treatment progress. Patients who completed the required number of CGC therapy units were considered for inclusion in the present analysis.

All relevant data pertaining to patients’ care and medical treatment were documented and recorded systematically and used regularly as the basis for interdisciplinary conferences, quality assurance measures, and billing calculations. Baseline demographic parameters as well as relevant information regarding patients’ morbidity and functional outcome were used for the current analysis: age, sex, medical comorbidities, information on short-term adverse events during hospitalization, and results of functional assessments on admission and discharge. Data regarding walking ability as assessed using the Timed Up and Go test on admission and discharge in particular were used for the current analysis. Data obtained from commonly used assessments in the geriatric field were also used for the current investigation (Barthel Index, Tinetti Geriatric Assessment, Geriatric Depression Scale, Mini-Mental State Examination (MMSE)) [10,11,12,13,14,15,16,17].

### 2.2. Assessment of Walking Ability (Timed Up and Go Test, TuG)

The TuG is a widely used simple test to assess persons’ mobility, verifying both the dynamic and static balance. TuG assesses the time a person needs to rise from a chair, walk three meters, turn around, walk back, and sit down again [33]. Based on the time required to complete the test, we categorized the results into 5 classes: (5) no walking ability at all; (4) >30 s needed to complete the test; (3) 20–29 s needed to complete the test; (2) 10–19 s to complete the test and (1) <10 s needed to complete the test. TuG assessments considered for our analysis were performed prior to and after CGC; for patients with fractures TuG assessments performed prior to CGC were considered. Based on the calculated difference between admission and discharge, we categorized walking ability after CGC into three classes: unchanged, improved, and worsening. For the analysis of factors associated with improvement in walking ability, patients with a positive value (≥1) for the difference between TuG on discharge and TuG on admission were compared to those with a corresponding difference ≤ 0.

### 2.3. Statistical Analyses

All data for continuous variables were expressed as median and interquartile ranges. Categorical variables were reported as frequencies and percentages. Normal distribution was verified using Kolmogorov–Smirnov’s one-sample test. Nonparametric data were analyzed by applying a two-tailed Mann–Whitney U-test. Fisher’s exact test was used to compare relative frequencies. Statistical analyses were performed using the SPSS software (version 22.0, IBM Corporation, Armonk, NY, USA).

### 2.4. Ethical Approval

The study was reviewed by the local ethical committee, which gave approval for the analysis of data (protocol number: 2019-517-f-S).

## 3. Results

Out of 1,263 patients hospitalized in our department, 1,099 patients underwent CGC and were included in the analysis (median age: 83.1 years (IQR 79.0–87.8 years); 64.1% were female). Of these, 300 patients (27.3%) were referred from a surgical department where they had received acute treatment after suffering a bone fracture. The majority of these patients (*n* = 168, 56%) had a fracture of the lower extremities (Figure 1). The remaining 799 patients underwent CGC for other medical reasons. Of the 1099 patients treated with CGC, 36 patients (3.3%) died during hospitalization. Results including co-morbidities and adverse events are summarized in Table 1.

**Figure 2:**
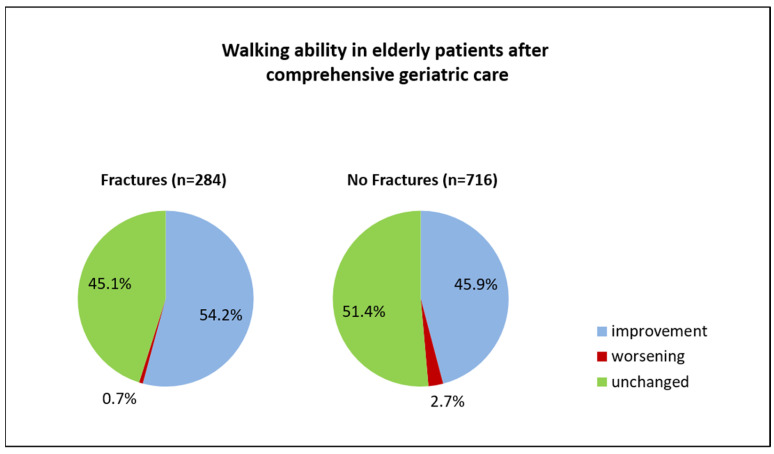
Patient outcomes, fractures versus no fractures.

An improvement in the TuG test after CGC was found in 54.2% of patients with fractures, compared to just 45.9% of patients without fractures (Figure 2). Comparing the TuG values on admission versus those measured on discharge, an improvement from median 5 (IQR 3–5) to median 3 (IQR 3–5) was noted in patients with fractures (*p* = 0.001). Patients with fractures (*n* = 300) were older than those without (*n* = 799), (median 85.6 vs. 82.4 years, *p* = 0.001) (Table 1). Higher frequencies of osteoporosis and dementia were detected in patients with fractures (dementia: 26.0% versus 18.7%, *p* = 0.007; osteoporosis: 18.0% versus 9.1%, *p* = 0.001). Patients with fractures had a median hospital stay of 17 days for CGC (IQR: 16–19 days) versus a median stay of 16 days (IQR: 16–19 days) for medically ill patients (*p* = 0.014) (Table 1).

**Figure 3:**
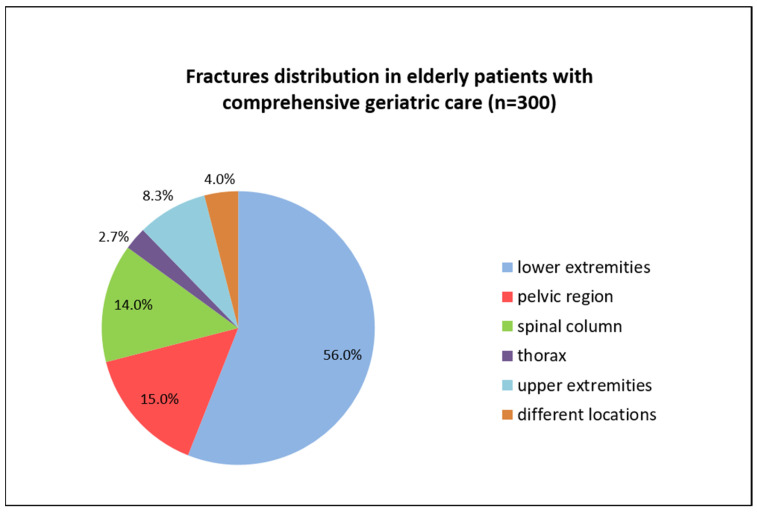
Distribution of fractures in elderly patients receiving comprehensive geriatric care.

In patients with fractures, the most frequently affected region was the lower extremity (56%) followed by the pelvic region (15%). The different fracture locations are summarized in Figure 3.

Patients completing the Timed Up and Go test 5 classes were categorized into five groups: (5) no walking ability at all; (4) >30 s needed to complete the test; (3) 20–29 s needed to complete the test; (2) 10–19 s needed to complete the test; and (1) <10 s needed to complete the test.

With regard to short-term adverse events, both groups had comparable proportions, apart from reported diffuse pain (fracture group 32.3% vs. non-fracture group 24.7%, *p* = 0.0012) (Table 1). The functional outcome on discharge (Barthel Index) was median 55 in the fracture group (IQR: 40–75) versus median 60 (IQR: 45–80) in the group without fractures (*p* = 0.030) (Table 1). Patients with fractures were discharged to regular care in 99% of cases versus 97.5% in those without fractures (*p* = 0.156) (Table 1).

**Figure 4:**
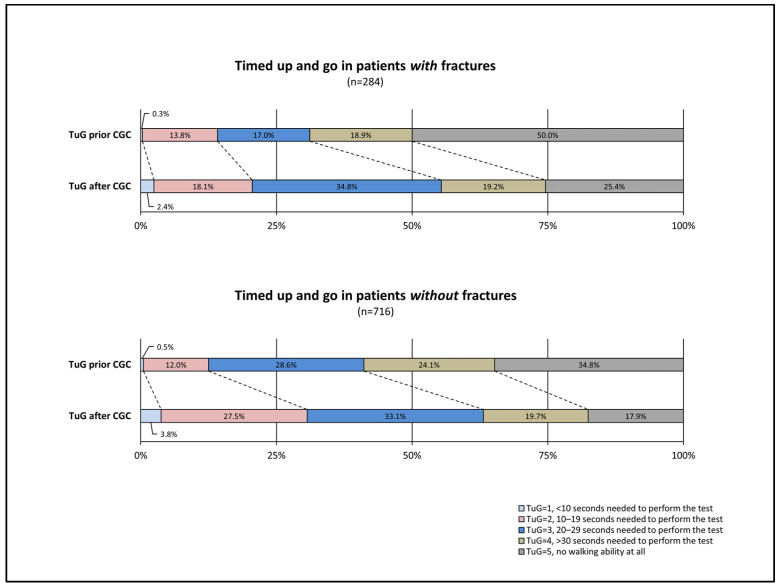
Walking ability in elderly patients prior to versus after comprehensive geriatric care. CGC = Comprehensive Geriatric Care; TuG = Timed Up and Go assessment. In patients with fractures, a TuG test result ≤ 4 prior CGC was detected in 50% of the cases, implicating that the other 50% were not able to walk at all. After the procedure of CGG, in 74.6% of the patients with fractures, a TuG result ≤ 4 was obtained. In patients without fractures, a TuG result ≤ 4 was detected in 65.2% of the cases; this value increased after the procedure of CGC to 82.1%.

A complete assessment on admission and discharge was identified in 284 patients with fractures (out of 300 patients, 94.7%) and in 716 patients without fractures, respectively (out of 799 patients, 89.6%). In patients with fractures, a TuG test result ≤ 4 prior CGC was detected in 50% of the cases, implicating that the other 50% were not able to walk at all. After the procedure of CGG, in 74.6% of the patients with fractures, a TuG result ≤ 4 was obtained. In patients without fractures, a TuG result ≤ 4 was detected in 65.2% of the cases; this value increased after the procedure of CGC to 82.1%. Results are summarized in Figure 4.

Fracture patients with improvement in walking ability (*n* = 154) were median 84.9 years (IQR: 81.1–89.4) old versus median 86.4 years (IQR: 81.2–90.7) in those without improvement (Table 2). Barthel Index and Tinetti Assessment values on admission were higher in the group of patients with fractures who demonstrated an improvement in walking ability after CGC than in fracture patients without improvement (Barthel index: median 45 (IQR: 35–55) versus 35 (IQR: 20–50); Tinetti: median 9 (IQR: 4–14.25) versus 5 (IQR: 0–13)) (Table 2). In the subgroup of patients with fractures, the percentage of individuals with dementia was higher in the subgroup without improvement in TuG after CGC than in the group with improvement (improvement: 21.4% versus no improvement: 31.5%; *p* = 0.058). Among fracture patients, no difference in hospital stay for CGC was noted between those with documented improvement in walking ability and those without (both: median 17 days, IQR: 16–19 days, *p* = 0.895) (Table 2).

## 4. Discussion

Our findings indicate that, after CGC, walking ability might improve in more than 50% of older patients who have suffered a fracture, while this is the case in just 45% of medically ill patients. The absolute frequency of patients who experienced a deterioration in walking ability was low in both fracture and non-fracture groups (0.7% and 2.7%). In patients with fractures, the better the initial functional status, the better the walking ability after CGC.

Although positive effects of CGC have previously been shown in general populations of older individuals, most of these investigations focused more on surgical patients and targeted the prevention of delirium after surgery [4,6,27,34]. In one recent study, Thingstad and co-workers demonstrated in a randomized trial that older patients with hip fractures had a better outcome in terms of walking ability after CGC than those receiving regular care [35]. However, the small sample size and the selection of patients in good initial condition might weaken the conclusions that can be drawn from this study [35]. By contrast, our study group comprises a considerable number of patients treated with CGC and also included immobilized patients, and therefore reflects real-world conditions. We were able to demonstrate a significant improvement in walking ability in both non-fracture and fracture patients. While more than half of patients in the latter group were unable to walk at all when referred to our department, 75% of them were able to perform the TuG test within 29 s or under after CGC. The proportion of patients with improvement in walking ability was slightly higher in the fracture group (54.2% versus 45.9%), indicating that such patients might benefit more from the treatment. This result is remarkable considering that the median age in the fracture group was higher than that in the non-fracture group. Although of younger age, the group of non-fracture medically ill patients had a higher proportion of male individuals, comorbidities, and vascular risk factors. As these factors indicate an increased general disease burden and a higher degree of frailty, the obvious selection bias towards healthier patients in the fracture group may go some way towards explaining the differences in both groups [36]. After CGC, a considerable proportion of patients maintained their walking ability, with deterioration observed in just a small number of cases. In the particular setting of a geriatric inpatient department, our study, therefore, proved that CGC was beneficial for the vast majority (>95%) of the patients included.

The analysis within the subgroup of patients with fractures revealed the factor documented diagnosis of dementia as being negatively associated with an improvement in walking ability after CGC. In line with this result, a recent publication has described cognitive decline as interfering with rehabilitation after surgery, especially by influencing the outcome of walking ability [37]. However, MMSE scores in our study may not support this observation. Although the factor of dementia was negatively associated with improvements in walking ability, there were no significant differences between the two groups in terms of MMSE scores. At this point, the missing MMSEs for around 20% of the patients in our study need to be acknowledged as potentially confounding the result. On the other hand, deterioration of MMSE performance might not correlate with the diagnosis of dementia.

By contrast, a better baseline status regarding activities of daily life was positively associated with an improvement in walking ability; higher Barthel Index and Tinetti assessment scores on admission were detected in the subgroup with improvement in walking ability after CGC. In line with previous investigations, our results indicated that a better functional status prior to suffering a fracture facilitated greater benefits following CGC [38]. In this context, our study presents important information underlining the relevance of functional assessments after surgery in order to select the most appropriate candidates for CGC.

The two major strengths of this study are the number of patients who had completed CGC available for analysis with detailed documentation of parameters. This study also has several limitations which should be acknowledged at this point. First, the selection bias needs to be taken into account when interpreting our results, as the geriatric pre-assessment might tend to select patients who are expected to benefit most from the procedure. Second, no control group (e.g., patients in a regular ward who did not undergo CGC) was available for comparison. A further limitation of the present study is the single-center design, which means that no extrapolation to other clinics or general populations is possible. Moreover, 5.3% of patients with fractures were missing a completed TuG assessment, so these patients needed to be excluded from the analysis. However, our study demonstrated concisely in a real-world environment that patients benefit from the procedure of CGC, especially surgical patients after suffering a fracture.

## 5. Conclusions

CGC in specialized geriatric departments improves walking ability in more than half of the participants. This may apply to all patients, but especially to those with fractures and of older age. A better functional status prior to CGC favors the improvement of walking ability after CGC, and this tendency is particularly pronounced in older patients with fractures.

## Figures and Tables

**Table 1:** Elderly patients with versus without fractures treated in a structured geriatric setting.

	Total Group (*n* = 1099)	Fractures (*n* = 300)	No Fractures (*n* = 799)	*p* *
**Age** (median, IQR, years)	83.1 (79.0–87.8)	85.6 (81.1–89.6)	82.4 (78.3–86.9)	0.001
**Age ≥ 80 years**	754 (68.6%)	239 (79.7%)	515 (64.5%)	0.001
**Sex**				
Female	704 (64.1%)	219 (73.0%)	485 (60.7%)	0.001
Male	395 (35.9%)	81 (27.0%)	314 (39.3%)	
**Co-morbidities**				
Hypertension	853 (77.6%)	246 (82.0%)	607(76.0%)	0.035
Diabetes mellitus	337 (30.7%)	69 (23.0%)	268 (33.5%)	0.001
Heart failure	258 (23.5%)	66 (22.0%)	192 (24.0%)	0.523
Renal insufficiency	382 (34.8%)	86 (28.7%)	296 (37.0%)	0.010
Coronary heart disease	281 (25.6%)	61 (20.3%)	220 (27.5%)	0.016
Peripheral artery disease	59 (5.4%)	9 (3.0%)	50 (6.3%)	0.035
Atrial fibrillation	388 (35.3%)	93 (31.0%)	295 (36.9%)	0.076
Chronic pulmonary artery disease	108 (9.8%)	18 (6.0%)	90 (11.3%)	0.009
Dementia	226 (20.6%)	78 (26.0%)	148 (18.5%)	0.007
Parkinson’s disease	62 (5.6%)	18 (6.0%)	44 (5.5%)	0.770
Previous stroke	90 (8.2%)	30 (10.0%)	60 (7.5%)	0.177
Osteoporosis	127 (11.6%)	54 (18.0%)	73 (9.1%)	0.001
Vitamin B deficiency ^ǁ^	484 (%)	151 (50.3%)	333 (41.7%)	0.012
**Short-term adverse events while hospitalized**				
Diffuse Pain	294 (26.8%)	97 (32.3%)	197 (24.7%)	0.012
Delirium	58 (5.3%)	14 (4.7%)	44 (5.5%)	0.651
Pneumonia	64 (6.1%)	15 (5.0%)	49 (6.1%)	0.564
Urinary tract infection	161 (14.3%)	47 (15.7%)	114 (14.6%)	0.566
Dizziness	82 (7.5%)	15 (5.0%)	67 (8.4%)	0.070
Deep vein thrombosis	5 (0.5%)	1 (0.3%)	4 (0.5%)	0.999
Pulmonary emboli	5 (0.5%)	1 (0.3%)	4 (0.5%)	0.999
Electrolyte imbalance	410 (39.0%)	98 (32.7%)	312 (37.3%)	0.059
Hypokalemia	103 (9.4%)	26 (8.7%)	77 (9.6%)	0.728
Hyponatremia	354 (32.2%)	82 (27.3%)	272 (34.0%)	0.036
**Functional assessment on admission**				
Barthel Index (median, IQR)	45 (30–60)	40 (30–50)	45 (30–60)	0.001
Tinetti Geriatric Assessment (median, IQR)	11 (12–16)	8 (1–14)	12 (4–17)	0.001
Geriatric Depression Scale (median, IQR)	3 (1–6)	3 (1–6)	3 (1–6)	0.844
Geriatric Depression Scale > 5	302 (27.7%)	83 (27.8%)	219 (27.6%)	0.999
Timed Up and Go (median, IQR)	4 (3–5)	5 (3–5)	4 (3–5)	0.001
MMSE (median, IQR) (*n* = 812)	26 (21–28)	25 (19–28)	26 (21–28)	0.282
**Patients deceased during hospitalization**	36 (3.3%)	6 (2.0%)	30 (3.8%)	0.183
**Functional assessment on discharge**	n = 1000	n = 284	n = 716	
Barthel Index (median, IQR)	60 (40–80)	55 (40–75)	60 (45–80)	0.030
Tinetti Geriatric Assessment (median, IQR)	16 (6–20)	14 (8–19)	16 (9–21)	0.006
Timed Up and Go (median, IQR)	3 (2–4)	3 (3–5)	3 (2–4)	0.001
Timed Up and Go improvement	483 (48.3%)	154 (54.2%)	329 (45.9%)	0.021
**Discharging modus**	n = 1063	n = 294	n = 769	
Regular discharge ^‡^	1041 (97.9%)	291 (99%)	750 (97.5%)	0.156
Referral to other department	22 (2.1%)	3 (1.0%)	19 (2.5%)	0.156
Length of hospital stay for patients with CGC ^¥^ (median, IQR, days)	17 (16–19)	17 (16–19)	16 (16–19)	0.014

* Interquartile range. ^ǁ^ Includes intracerebral and subarachnoid hemorrhages, subdural hemorrhages, and unspecified head injuries. ^¥^ Comprehensive Geriatric Care, ^‡^ includes home care with and without assistance, nursing home.

**Table 2:** Improvement in walking ability (defined as TuG score difference ≥ 1 between discharge and admittance) in elderly patients with fractures after treatment in a structured geriatric setting.

	Total Group (*n* = 284)	Improvement in Walking Ability (*n* = 154)	No Improvement in Walking Ability (*n* = 130)	*p* *
**Age** (median, IQR, years)	85.6 (81.1–89.9)	84.9 (81.1–89.4)	86.4 (81.2–90.7)	0.250
**Age ≥ 80 years**	228 (80.3%)	124 (80.5%)	104 (80.0%)	0.999
**Sex**				
Female	210 (73.9%)	113 (73.4%)	97 (74.6%)	0.892
Male	74 (26.1%)	41 (26.6%)	33 (25.4%)	
**Co-morbidities**				
Hypertension	231 (81.3%)	123 (79.9%)	108 (83.1%)	0.534
Diabetes mellitus	66 (23.2%)	33 (21.4%)	33 (25.4%)	0.482
Heart failure	60 (21.1%)	32 (20.8%)	28 (21.5%)	0.885
Renal insufficiency	82 (28.9%)	47 (30.5%)	35 (26.9%)	0.515
Coronary heart disease	56 (19.7%)	31 (20.1%)	25 (19.2%)	0.882
Peripheral artery disease	9 (3.2%)	5 (3.2%)	4 (3.1%)	0.999
Atrial fibrillation	87 (30.6%)	47 (30.5%)	40 (30.8%)	0.999
Chronic pulmonary artery disease	16 (5.6%)	8 (5.2%)	8 (6.2%)	0.799
Dementia	74 (26.1%)	33 (21.4%)	41 (31.5%)	0.058
Parkinson’s disease	18 (6.3%)	8 (5.2%)	10 (7.7%)	0.467
Previous stroke	27 (9.5%)	12 (7.8%)	15 (11.5%)	0.314
Osteoporosis	53 (18.7%)	35 (22.7%)	18 (13.8%)	0.067
Vitamin B deficiency ^ǁ^	144 (50.7%)	75 (48.7%)	69 (53.1%)	0.477
**Short-term adverse events while hospitalized**				
Diffuse pain	91 (32.0%)	49 (31.8%)	42 (32.3%)	0.999
Delirium	14 (4.9%)	7 (4.5%)	7 (5.4%)	0.789
Pneumonia	11 (3.9%)	5 (3.2%)	6 (4.6%)	0.557
Urinary tract infection	45 (15.8%)	25 (16.2%)	20 (15.4%)	0.872
Dizziness	14 (4.9%)	10 (6.5%)	4 (3.1%)	0.272
Deep vein thrombosis	1 (0.4%)	0 (0%)	1 (0.8%)	0.458
Pulmonary emboli	1 (0.4%)	0 (0%)	1 (0.8%)	0.458
Electrolyte imbalance	91 (32.0%)	50 (32.5%)	41 (31.5%)	0.899
Hypokalemia	25 (8.8%)	16 (10.4%)	9 (6.9%)	0.401
Hyponatremia	75 (26.4%)	41 (26.6%)	34 (26.2%)	0.999
**Functional assessment on admission**				
Barthel index (median, IQR)	40 (30–50)	45 (35–55)	35 (20–50)	0.001
Tinetti on admission (median, IQR)	8 (1–14)	9 (4–14.25)	5 (0–13)	0.001
Geriatric depression scale (median, IQR)	3 (1–6)	4 (1.75–6)	3 (0–6)	0.134
Geriatric depression scale >5	77 (27.1%)	42 (27.3%)	35 (26.9%)	0.999
Timed up and go (median, IQR)	5 (3–5)	5 (4–5)	5 (3–5)	0.151
MMSE (median, IQR) (*n* = 226)	25 (19–28)	26 (19–28)	25 (19–28)	0.779
**Length of hospital stay for CGC ^¥^ (median, IQR, days)**	17 (16–19)	17 (16–19)	17 (16–19)	0.895

* Interquartile range. ^ǁ^ Includes intracerebral and subarachnoid hemorrhages, subdural hemorrhages, and unspecified head injuries. ^¥^ Comprehensive Geriatric Care.

## Data Availability

For data disposition and sharing, please contact: christian.tanislav@diakonie-sw.de.

## Abbreviations

CGC	Comprehensive Geriatric Care
TuG	Timed Up and Go test
IQR	interquartile range
MMSE	Mini-Mental State Evaluation
